# Metabolic Profiling of the Protozoan Parasite *Entamoeba invadens* Revealed Activation of Unpredicted Pathway during Encystation

**DOI:** 10.1371/journal.pone.0037740

**Published:** 2012-05-25

**Authors:** Ghulam Jeelani, Dan Sato, Afzal Husain, Aleyla Escueta-de Cadiz, Masahiro Sugimoto, Tomoyoshi Soga, Makoto Suematsu, Tomoyoshi Nozaki

**Affiliations:** 1 Department of Parasitology, National Institute of Infectious Diseases, Shinjuku, Tokyo, Japan; 2 Department of Biochemistry and Integrative Medical Biology, School of Medicine, Keio University, Shinjuku, Tokyo, Japan; 3 Institute for Advanced Biosciences, Keio University, Tsuruoka, Yamagata, Japan; 4 Department of Parasitology, Graduate School of Medicine, Gunma University, Maebashi, Japan; 5 Graduate School of Life and Environmental Sciences, University of Tsukuba, Tsukuba, Ibaraki, Japan; Hospital for Sick Children, Canada

## Abstract

Encystation, which is cellular differentiation from the motile, proliferative, labile trophozoite form to the dormant, resistant cyst form, is a crucial process found in parasitic and free-living protozoa such as *Entamoeba*, *Giardia*, *Acanthamoeba*, and *Balamuthia*. Since encystation is an essential process to deal with the adverse external environmental changes during the life cycle, and often integral to the transmission of the diseases, biochemical understanding of the process potentially provides useful measures against the infections caused by this group of protozoa. In this study, we investigated metabolic and transcriptomic changes that occur during encystation in *Entamoeba invadens*, the reptilian sibling of mammal-infecting *E. histolytica*, using capillary electrophoresis-tandem mass spectrometry-based metabolite profiling and DNA microarray-based expression profiling. As the encystation progressed, the levels of majority of metabolites involved in glycolysis and nucleotides drastically decreased, indicating energy generation is ceased. Furthermore, the flux of glycolysis was redirected toward chitin wall biosynthesis. We found remarkable temporal increases in biogenic amines such as isoamylamine, isobutylamine, and cadaverine, during the early period of encystation, when the trophozoites form large multicellular aggregates (precyst). We also found remarkable induction of γ-aminobutyric acid (GABA) during encystation. This study has unveiled for the first time the dynamics of the transcriptional and metabolic regulatory networks during encystation, and should help in better understanding of the process in pathogenic eukaryotes, and further development of measures controlling infections they cause.

## Introduction

Differentiation or developmental stage conversion is ubiquitous in all living organisms. For many pathogenic protozoa, it is essential for their survival and transmission. Several parasitic protozoa such as *Entamoeba histolytica* [1] and *Giardia lamblia* [2], which cause diarrhea, and free-living protozoa including *Balamuthia mandrillaris*, which causes granulomatous encephalitis [3], and *Acanthamoeba castellanii*, responsible for amoebic keratitis and encephaltitis [4], present two morphologically distinct stages in their life cycle: the motile, proliferative, labile trophozoite form, which inhabits the mammalian hosts and is responsible for pathogenesis, and the resistant cyst, which is protected by a cell wall that allows survival under the adverse external environment, and responsible for transmission [5]. The encystation process has been attracting attention not only from the viewpoint of disease transmission, but also as a model for differentiation. Therefore, elucidation of the encystation process, both at the metabolic and transcriptomic levels should lead to a better understanding of the process and, therefore, to the possibility of better control measures against infectious diseases the parasites cause.

The intestinal parasite *Entamoeba histolytica*, the causative agent of amoebiasis, is estimated to infect 50 million people annually, mainly in developing countries, where it is a major source of morbidity and mortality [6]. No axenic *in vitro* system currently exists for studying encystation of the human-infecting parasite *Entamoeba histolytica*; however, such a system exists for *E. invadens*, a parasite of reptiles. These two organisms have the same two-stage life cycle and pathogenic potential toward their vertebrate hosts [7], and form quadrinucleated, chitinaceous cysts with osmotically resistant cyst walls [8]. *E. invadens* will readily encyst *in vitro* in response to carbon source deprivation [9], hypoosmotic shock [10], or a combination of the two stimuli [11].

As most of current drugs against protozoa target metabolism, it is critical to understand the structure and dynamics of the parasite metabolic network during encystation. Indirect approaches to reconstructing the metabolic network, by comparative genomics and enzymological studies of individual enzymes, are at the best incomplete and face major obstacles in highly divergent organisms such as parasitic protozoa. Global metabolomics is a new and powerful technology that provides a relatively complete picture of the metabolism in biological systems and has recently been applied to a wide variety of important problems [12]–[15]. We decided to apply this approach to understand the basis of the changes in cellular metabolism that occur during encystation. To better understand the relationship between gene expression and metabolites levels, we also analyzed the mRNA expression profile of the enzymes involved in the formation or utilization of these metabolites.

## Results and Discussion

### Overall strategy of metabolome and transcriptome analyses of encystation

**Figure 1:**
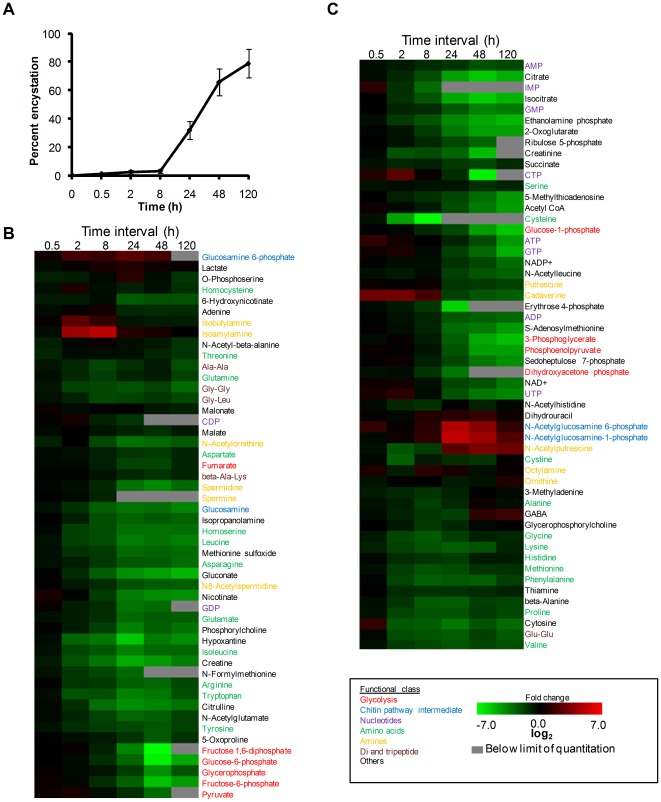
Change in metabolites during encystation. (**A**) Kinetics of encystation. The percentages of the amoebae resistant to 0.05% sarkosyl during encystation are shown. (**B and C**) Heat map produced by hierarchical clustering of metabolites profiles obtained from CE-TOFMS analysis. Rows correspond to metabolites and columns correspond to time intervals. Shown are 104 metabolites detected during encystation. Metabolites levels are expressed as log_2_ of the fold change with respect to time 0 h. Shades in red and green indicate an increase and decrease of metabolites, respectively, according to the scale bar shown at the bottom.

**Figure 2:**
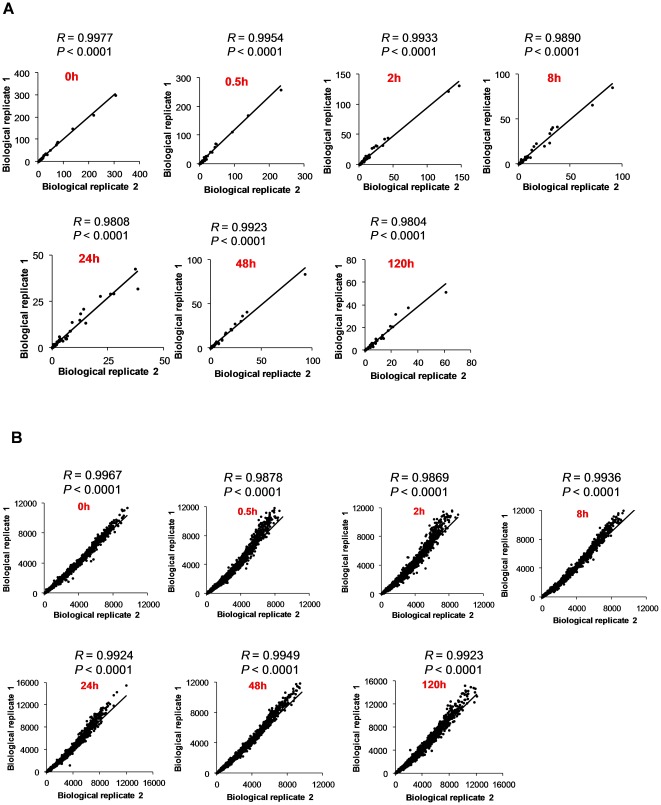
Correlation between two biological replicates. The correlation of the levels of metabolites in metabolomic analysis (**A**) and transcripts in DNA microarray analysis (**B**) between first and second biological replicates at different time points during encystation is shown. The Pearson correlation coefficient and their P-values (two tailed) were calculated using GraphPad prism version 5.04.

In vitro encystation of *E. invadens* was carried out using the 47% LG medium lacking glucose [11]. Under these conditions, approximately 80% of the trophozoites differentiated into the sarkosyl-resistant cysts within 120 h (Figure 1A). We also verified the cyst formation by calcofluor staining, and showed that the percentage of the sarkosyl-resistant amoebae and that of the calcofluor-stained amoebae were comparable (data not shown). Metabolite extracts were prepared from the cell harvested at different time points during encystation (0, 0.5, 8, 24, 48, and 120 h). The capillary electrophoresis-time-of-flight mass spectrometry (CE-TOFMS) systems in cation and anion modes were used to identify the peaks [16]. The main peaks were identified and quantified with metabolite standards by matching the closest m/z values and normalized migration times for further statistical comparisons and interpretations. All data presented were normalized by cell number (per 10^6^ cells) as it is the method commonly used and practically accepted in most of studies [17], [18]. In addition, it is not practically possible to normalize metabolite data with cell volume, because the population during encystation is polymorphic and heterogeneous (i.e., a mixture of trophozoites and cysts with different proportions at different time points). However, one should note that the trophozoites and the cyst slightly differ in size (the diameter of usual trophozoites and cysts of *E. histolytica* ranges 13–20 and 11–14 µm, respectively [19]). Therefore, our data need to be carefully interpreted as the metabolite concentrations in cysts tend to be underestimated (potentially >2 fold). However, most, if not all, changes in metabolites presented here largely reflect changes in intracellular concentrations, but not in cell volume. The identified metabolites and their quantities are listed in Dataset S1. We identified more than 100 intermediary metabolites, which include amino acids, nucleotides, biosynthetic precursors, and central carbon metabolism intermediates (Figure 1B and C). To validate the reproducibility of the results, we compared the metabolomic and transcriptomic data from the two biological replicates at different time points during encystation. A nearly perfect correlation between the first and second replicates was observed (Figure 2).

Using hierarchical clustering analysis [20], we grouped metabolites by their temporal profiles and identify those that exhibited similar changes in the profile during encystation (Figure 1B and C). For example, clustering analysis revealed that the metabolites that are involved in chitin biosynthetic pathway displayed highly similar profiles (see below). Moreover, other metabolites known to be in common biochemical pathways such as polyamine metabolism showed similar temporal abundance profiles (see below). To correlate changes in metabolite levels with changes in transcript levels, we also globally examined gene expression by DNA microarray analysis (Dataset S2).

### Glycolysis and chitin biosynthetic pathway

**Figure 3:**
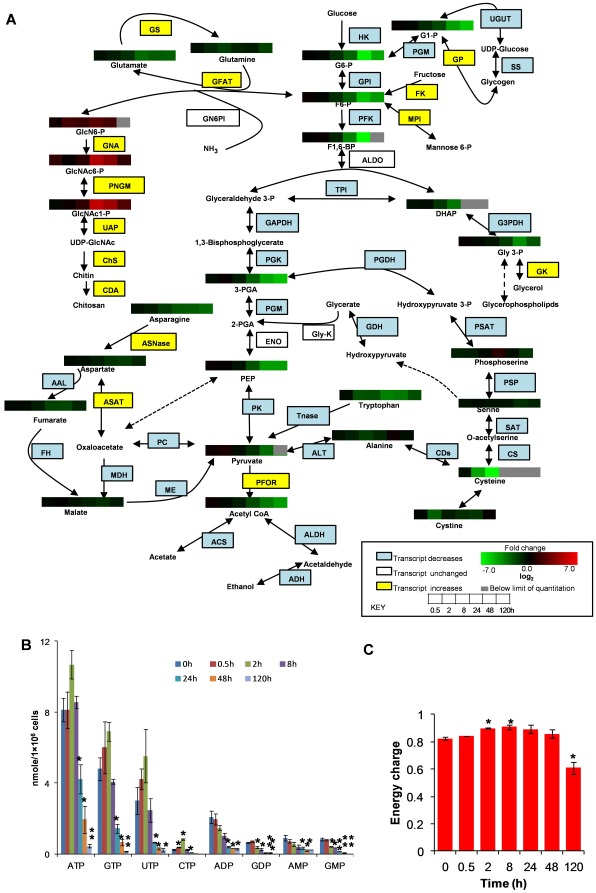
Central carbon metabolism during encystation. (**A**) Alterations in the levels of metabolites involved in central carbon metabolism during encystation. The metabolite levels represented by heat map strip and the transcript levels shown in rectangles during encystation are superimposed on a metabolic pathway map that includes glycolysis, chitin biosynthesis, serine biosynthesis, and other related pathways. Metabolites levels are expressed as log_2_ of the fold change with respect to time 0 h. Shades in red and green indicate an increase and decrease of metabolites, respectively, according to the scale bar. The enzymes are shown in yellow or blue in case where their transcript levels increased or decreased by >3 fold relative to time 0 h, respectively. The entire quantitative metabolite data, as well as the indicator of statistical significance are given in Dataset S1. Abbreviations are: G 6-P, glucose 6-phosphate; G1-P, glucose 1-phosphate; F6-P, fructose 6-phosphate; F1,6-BP, fructose 1,6-biphosphate; DHAP, dihydroxy acetone phosphate; Gly 3-P, glycerol 3-phosphate; 3-PGA, 3-phosphoglycerate; PEP, phosphoenolpyruvate; GlcN6P, glucosamine 6-phosphate; GlcNAc6-P, N-acetylglucosamine 6-phosphate; GlcNAc1-P, N-acetyl glucosamine 1-phosphate; UDP-GlcN6P, UDP-glucosamine-6-phosphate. The full names of the enzymes denoted by abbreviations, the basal expression levels at time 0 h, and the fold changes relative to 0 h at different time points during encystation can be found in supplementary dataset S2. (**B**) The average concentration of the nucleotides at various time points during encystation. Data are depicted as means ± S. D. Statistical comparisons were made by Student's t test (* P<0.05, ** P<0.01). (**C**) Adenylate energy charge of the cell, which is calculated by the equation, [(ATP)+1/2(ADP)]/[(ATP)+(ADP)+(AMP)] during encystation. Data are shown as means ± S.D. Statistical comparisons were made by Student's t test (* P<0.05, ** P<0.01).

Glucose metabolism plays a pivotal role in cellular metabolism to produce energy and precursors of nucleotides and fatty acids [21]. Several lines of evidence suggest that this parasite relies solely on glycolysis for ATP supply, as it is devoid of the Krebs cycle and oxidative phosphorylation [22], [23]. We observed that the levels of various glycolytic pathway intermediates, including glucose-6-phosphate and fructose-6-phosphate, were significantly depleted during encystation (Figure 3A). While transcript levels of many genes involved in glycolysis, and other pathways also changed during encystation (Dataset S2), the metabolites involved in chitin biosynthesis were remarkably increased after 8 h of induction, suggesting that the flux of the glycolysis was redirected toward the chitin biosynthetic pathway (Figure 3A). Chitin is the major component of the cyst walls of protozoan parasites [24] including *E. histolytica* [25], *G. lamblia* [26], *B. mandrillaris* [27], and *A. castellanii* [28]. As chitin does not occur in vertebrates, its synthetic pathway represents an excellent parasite-specific target to develop new chemotherapeutics, as proposed [29]. We found that gene expression of all the enzymes involved in chitin wall biosynthesis also increased (Figure S1).

The transcript level of glucosamine-fructose-6-phosphate aminotransferase (GFAT), which is the first and rate-limiting enzyme [30] of the chitin biosynthetic pathway, increased during encystation, whereas that of glucosamine-6 phosphate isomerase (GN6PI) remained unchanged (Figure S1), suggesting that *Entamoeba* prefers glutamine to NH_3_ as an ammonia donor for the synthesis of chitin wall. Interestingly, glutamine synthase (GS) is also upregulated during encystation (Figure S1). GS catalyses the formation of glutamine from ammonium ion and glutamate. Thus, in order to meet the demands of glucosamine for chitin biosynthesis, GS seems to be co-up-regulated with GFAT to provide enough amino group donors for GFAT. The co-up-regulation of GFAT and GS may be linked to a different hypothetical role of the peritrophic matrix, namely elimination of toxic ammonium ions, which are released by utilization of arginine during encystation [5]. As reported previously, GFAT activity was sensitive to feedback inhibition by UDP-GlcNAc, the end product of the hexosamine pathway, and is modulated by cAMP-dependent protein kinase A [31]. GFAT has attracted the interest of several research groups [32] as it offers potential target for antibacterial and antifungal agents.

Expression of one of three genes encoding chitin deacetylase (CDA) (EIN_058630) was upregulated during encystation (Dataset S2), which led us to hypothesize that this protein deacetylates chitin to form chitosan (Figure 3A), which is a mixture of *N*-acetylglucosamine and glucosamine. Chitosan, which has a positive charge, is a major component of spore walls of *Saccharomyces cerevisiae* and lateral walls of *Mucor rouxii* [33]. The chitosan also has another important feature: the ability to inhibit the glycolytic pathway in cancerous cells [34].

We also found that the transcripts of the enzymes involved in glycogen/starch biosynthesis decreased, whereas those involved in their decomposition increased (Figure S1). This observation led us to propose that during encystation the glucose which is stored in the form of glycogen/starch may be redirected toward chitin synthesis. Apart from the chitin wall, we also found that the level of O-phosphoserine, which is an intermediate of serine biosynthesis, increased at 24 h (Figure 3A).

### Amino acid and nucleotides

Metabolomic analysis showed that most amino acids decreased during encystation, except alanine (Dataset S1). The observation is consistent with the premise that during encystation when all the glucose is used for the synthesis of chitin, various amino acids were used as an alternative energy source [35]. Interestingly, *Giardia* [36] and *Trichomonas* [37] were shown to produce alanine as a major end product of carbohydrate metabolism under anaerobic conditions [38], suggesting that carbohydrate metabolism during encystation in *Entamoeba* partially resembles that in *Giardia* and *Trichomonas*.

Expression of one of two ASAT (aspartate aminotransferase), which is involved in the conversion of aspartate to oxaloacetate (Dataset S2) (EIN_146870), increased by >400 fold during encystation, suggesting that asparagine as well as aspartate are used as an energy source when glucose is not available. It was previously reported that amino acids such as aspartate, asparagines, and arginine also serve as an energy source in anaerobic parasitic protists [39].

Consistent with the decrement of glycolytic intermediates and amino acids, energy content of the cell was drastically decreased during encystation. The levels of most nucleotides such as ATP, GTP, UTP, and CTP were transiently increased at 2 h, after which the level of all the nucleotides drastically decreased (Figure 3B). We also examined the average adenylate energy charge [40], which is calculated by the equation: [(ATP)+1/2(ADP)]/[(ATP)+(ADP)+(AMP)]. The energy charge in a growing organism is normally stabilized in the range of 0.8 to 0.95, and it is not greatly affected by the wide variations [41]. The energy charge of the cell notably decreased after 48 h during encystation (Figure 3C). The decrease in most purines and pyrimidines after 8 h was consistent with the previous observation for *Escherichia coli* that the nucleotide pools continuously decreased as cells moved from the exponential to stationary growth phase [42].

### Polyamine metabolism during encystation

**Figure 4:**
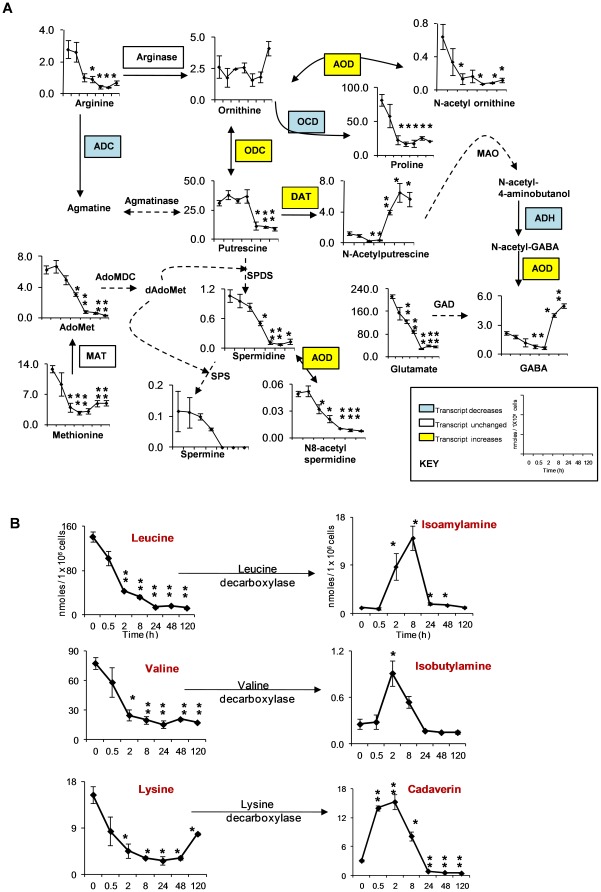
Polyamines and biogenic amines levels during encystation. (**A**) Metabolome and transcriptome data of the pathway involved in biosynthesis and catabolism of polyamines and related metabolites from arginine. The average concentrations of each metabolite (nmol/1×10^6^ cells) at time intervals (0, 0.5, 2, 8, 24, 48 and 120 h) are shown. The enzymes are shown in yellow or blue in case where their transcript levels increased or decreased by >3 fold relative to time 0 h, respectively. Abbreviations are: GABA, γ-aminobutyric acid; AdoMet, S-Adenosyl methionine; dAdoMet, S-adenosylmethioninamine. Solid lines represent the steps catalyzed by the enzymes whose encoding genes are present in the genome, whereas dashed lines indicate those likely absent in the genome. Abbreviations are: MAO, monoamine oxidase; AdoMDC, S-adenosylmethionine decarboxylase; SPDS, spermidine synthase; SPS, spermine synthase; GAD, glutamate decarboxylase. Data are shown as means ± S.D. Statistical comparisons were made by Student's t test (* P<0.05, ** P<0.01). (**B**) Level of some biogenic amines and its precursors during encystation. X-axis represents time in hours, whereas Y-axis represents the concentration of metabolites in nmoles/1×10^6^ cells. Data are represented as means ± S.D. Statistical comparisons were made by Student's t test (* P<0.05, ** P<0.01).

Aliphatic polyamines putrescine, spermidine, and spermine, occur ubiquitously and have important functions in the stabilization of cell membranes, biosynthesis of informing molecules, cell growth, and differentiation, as well as adaptation to osmotic, ionic, pH and thermal stress [43], [44]. The inhibition of polyamine metabolism has important pharmacological and therapeutic implications for the control of physiological processes, reproduction, cancer, and parasitic diseases [45], [46]. Enzymes for the synthesis of longer polyamines, spermidine and spermine, were not identified in the genome of *Entamoeba*. This is puzzling as spermidine is known to be required for the formation of hypusine on the essential initiation factor eIF5A in eukaryotes [47]. This amino acid modification is likely present in *E. histolytica* as deoxyhypusine synthase, the enzyme that uses spermidine to create deoxyhypusine on eIF5A, was identified in the genome [48]. Spermidine is therefore likely to be an essential trace nutrient for *E. histolytica* and therefore spermidine synthesis by *E. histolytica* cannot be ruled out. Our metabolomic data also revealed that beside putrescine, other polyamines like spermidine, spermine, and N^8^-acetylspermidine were present in proliferating trophozoites and the level of these metabolites decreased as encystation proceeded. These finding indicate that the amoeba utilizes these polyamines during encystation (Figure 4A). Our result is also consistent with the previous finding that polyamine levels decreased during encystment in *A. castellanii* [49]. The role of N^8^-acetylspermidine in polyamine metabolism remains unclear. N^8^-acetylspermidine is known to be converted back into spermidine by a deacetylating enzyme [50], suggesting a reversible sequestration of excessive spermidine. The level of N-acetylputrescine, which is derived from putrescine, increased during encystation (Figure 4A). Polyamine acetylation appears to be a component of a cellular mechanism involved in polyamine turnover and excretion [51], which could function as a means of reducing the intracellular polyamine concentration. In addition, storage of the acetylated (metabolically inactive) form of polyamines in the dormant cells (cysts) could provide the newly activated cells with a readily available source of polyamines for growth purposes. Another consequence of polyamine acetylation is to decrease the positive charge, which helps to displace them from anionic binding sites as well as to increase their lipophilic properties that may aid transport processes [51].

### Induction of GABA during encystation

Surprisingly, we found that at the later stage of encystation (48 and 120 h) the level of γ-aminobutyric acid (GABA) increased (Figure 4A). GABA is made from L-glutamate in a single reaction catalyzed by the enzyme glutamate decarboxylase, which is missing in the *Entamoeba* genome. However, a number of amino acid decarboxylases are encoded in the genome, and it is possible that some of these decarboxylases convert glutamate to GABA. It has been demonstrated that in other organisms, GABA is also made from an alternative putrescine pathway [52]. The concentration profile of N-acetylputrescine during encystation was similar to that of GABA; the increase of N-acetylputrescine slightly preceded that of GABA (Figure 4A). These data are consistent with the premise that GABA may be synthesized from N-acetylputrescine during encystation. However, further labeling experiments are required to verify the formation of GABA from either glutamate or putrescine. The role of GABA in *Entamoeba* is unclear. GABA has also been reported in other protozoan parasites like *Plasmodium falciparum* [53]. GABA is the major inhibitory neurotransmitter in mammalian central nervous system. This neurotransmitter has an ancient heritage as an intercellular signal and has been reported to induce terminal differentiation (sporulation) of *Dictyostelium discoideum*, a soil-living amoeba, through a GABA_B_ receptor [54]. In plants, GABA is thought to play a role in various stress responses, like heat stress, which leads to the six to ten-fold accumulation of GABA compared to an unstressed plant [55], sudden decrease in temperature [56], and water stress [57]. Rapid GABA accumulation in response to wounding was also indicated to play a role in plant defense against insects [58].

### Biogenic amines increases during early period of encystation

Biogenic amines are naturally occurring compounds, ubiquitous in animals and plants. They are low molecular weight organic bases, aliphatic (putrescine, cadaverine, isoamylamine, isobutylamine), heterocyclic (histamine and tryptamine), or aromatic (tyramine and phenylethylamine) [59]. These compounds are known to play important roles in normal mammalian physiology, like cell proliferation and differentiation [60]. We also found remarkable changes in biogenic amines such as cadaverine, isoamylamine, and isobutylamine (Figure 4B), which increased during the early period of encystation (0.5 to 8 h), when the trophozoites formed large multicellular aggregates (precyst), and then decreased when the precyst differentiated to the cyst. These three biogenic amines showed distinct kinetics during encystation, suggesting that these biogenic amines could play a specific role in distinct processes of encystation. Biogenic amines play an important role in induction of encystation in *Hartmannella vermiformis*, a nonpathogenic free-living amoeba that is the natural reservoir of *Legionella pneumophila*, the causative agent of Legionnaire's disease [61]. Biogenic amines such as cadaverine, isoamylamine and isobutylamine are the decarboxylated product of amino acids such as lysine, leucine, and valine. It was previously suggested that *E. invadens* encystation and *E. histolytica* cyst-like structure formation are induced by CO_2_ [62]. Therefore, it is conceivable that the CO_2_, which is released during the formation of biogenic amines, may also participate in inducing the encystation process.

### Conclusion and future directions

In summary, our metabolome and transcriptome analyses provided, for the first time in eukaryotes, global changes of metabolisms during a major differentiation process from trophozoites to cysts. Genomic reconstructions, which generally map genomic information of an individual organism onto the metabolic networks of well-studied model organisms, must be informed by direct experimental metabolic evidence. Otherwise, they likely fail to identify the best candidate pathways for drug targets. We found increase in the level of some biogenic amines as well as γ-aminobutyric acid (GABA) during encystation. Finally, further works are needed to characterize key metabolites and their functions responsible for the signal transduction pathways triggering encystation via the formation of GABA and biogenic amines.

## Materials and Methods

### *E. invadens* culture and encystation

Trophozoites of the *E. invadens* IP-1 strain were cultured axenically in BI-S-33 medium at 26°C. To induce encystation, 2-week-old *E. invadens* cultures were passaged in 47% LG medium lacking glucose [11] at approximately 6×10^5^ cells/ml. Amoebae were collected at various time points, and the formation of cysts was assessed by virtue of the resistance to 0.05% sarkosyl using 0.22% trypan blue to selectively stain dead cells. Cysts were also verified by cyst wall staining by incubating amoebae with calcofluor white (fluorescent brightener; Sigma-Aldrich) at room temperature.

### Metabolic extraction

Intracellular metabolites were extracted as described previously with some modifications [63]. Approximately 1.5×10^6^ *E. invadens* cells were harvested after 0, 0.5, 2, 8, 24, 48, and 120 h cultivation in 47% LG medium lacking glucose. The cells were immediately suspended in 1.6 ml of −75°C methanol to quench metabolic activity. Given the speed of metabolic reactions, quickly removing medium and immediately quenching metabolism is essential for obtaining reliable results. The selection of cold methanol as an extraction solvent was based on systematic studies in bacteria and yeast, which point to its providing relatively good extraction of a broad spectrum of metabolites [64]–[65], while avoiding marked metabolite decomposition and associated formation of decomposition products, which can themselves mimic metabolites [66]. To ensure that experimental artifacts such as ion suppression did not lead to misinterpretation of metabolite levels, internal standards, 2-(*N*-morpholino) ethanesulfonic acid, methionine sulfone, and D-camphor- 10-sulfonic acid were added to every sample. The samples were than sonicate for 30 second and then mixed with 1.6 ml of chloroform and 640 µl of deionized water. After vortexing, the mixture was centrifuged at 4,600× *g* at 4°C for 5 min. The aqueous layer (1.6 ml) was filtrated using an Amicon Ultrafree-MC ultrafilter (Millipore Co.) and centrifuged at 9,100× *g* at 4°C for ∼2 h. The filtrate was dried and preserved at −80°C until mass spectrometric analysis [67]. Prior to the analysis, the sample was dissolved in 20 µl of deionized water containing reference compounds (200 µM each of 3-aminopyrrolidine and trimesic acid).

### Instrumentation of capillary electrophoresis-time-of-flight mass spectrometry (CE-TOFMS)

CE-TOFMS was performed using an Agilent CE Capillary Electrophoresis System equipped with an Agilent 6210 time-of-flight mass spectrometer, Agilent 1100 isocratic HPLC pump, Agilent G1603A CE-MS adapter kit, and Agilent G1607A CE-ESI-MS sprayer kit (Agilent Technologies, Waldbronn, Germany). The system was controlled by Agilent G2201AA ChemStation software for CE. Data acquisition was performed by Analyst QS software for Agilent TOF (Applied Biosystems, CA, USA;MDS Sciex, Ontario, Canada).

### CE-TOFMS conditions for cationic metabolite analysis

Cationic metabolites were separated in a fused-silica capillary (50 µm i.d.×100 cm total length) filled with 1 M formic acid as the electrolyte [68]. Sample solution (∼3 nL) was injected at 50 mbar for 3 s, and a positive voltage of 30 kV was applied. The capillary and sample trays were maintained at 20°C and below 5°C, respectively. Sheath liquid composed of methanol/water (50% v/v) that contained 0.1 µM hexakis (2,2-difluorothoxy) phosphazene was delivered at 10 µL/min. ESI-TOFMS was operated in the positive ion mode. The capillary voltage was set at 4 kV and a flow rate of nitrogen gas (heater temperature 300°C) was set at 10 psig. For TOFMS, the fragmenter voltage, skimmer voltage, and octapole radio frequency voltage (Oct RFV) were set at 75, 50, and 125 V, respectively. An automatic recalibration function was performed using two reference masses of reference standards; protonated ^13^C methanol dimer (*m/z* 66.063061) and protonated hexakis (2,2-difluorothoxy) phosphazene (*m/z* 622.028963), which provided the lock mass for exact mass measurements. Exact mass data were acquired at the rate of 1.5 cycles/s over a 50 to 1,000 *m/z* range.

### CE-TOFMS conditions for anionic metabolite analysis

Anionic metabolites were separated in a cationic-polymer–coated COSMO(+) capillary (50 µm i.d.×110 cm) (Nacalai Tesque) filled with 50 mmol/L ammonium acetate solution (pH 8.5) as the electrolyte [69]. Sample solution (∼30 nL) was injected at 50 mbar for 30 s and a negative voltage of −30 kV was applied. Ammonium acetate (5 mmol/L) in 50% methanol/water (50% v/v) that contained 0.1 µmol/L hexakis (2,2-difluorothoxy) phosphazene was delivered as sheath liquid at 10 µL/min. ESI-TOFMS was operated in the negative ion mode. The capillary voltage was set at 3.5 kV. For TOFMS, the fragmenter voltage, skimmer voltage, and Oct RFV were set at 100, 50, and 200 V, respectively [70]. An automatic recalibration function was performed using two reference masses of reference standards: deprotonated ^13^C acetate dimer (*m/z* 120.038339) and acetate adduct of hexakis (2,2-difluorothoxy) phosphazene (*m/z* 680.035541). The other conditions were identical to those used for the cationic metabolome analysis.

### CE-TOFMS data processing

Raw data were processed using the in-house software Masterhands [71]. The overall data processing flow consisted of the following steps: noise-filtering, baseline-removal, migration time correction, peak detection, and integration of peak area from a 0.02 *m/z*-wide slice of the electropherograms. This process resembled the strategies employed in widely used data processing software for LC-MS and GC-MS data analysis, such as MassHunter (Agilent Technologies) and XCMS [72]. Subsequently, accurate *m/z* values for each peak were calculated by Gaussian curve fitting in the m/z domain, and migration times were normalized using alignment algorithms based on dynamic programming [73]. All target metabolites were identified by matching their *m/z* values and normalized migration times with those of standard compounds in the in-house library. All data presented were normalized by cell number (per 10^6^ cells).

### RNA isolation and affymetrix microarray hybridization

*E. invadens* cells were harvested after 0, 0.5, 2, 8, 24, 48 and 120 h cultivation in 47% LG medium lacking glucose and washed twice with phosphate buffer saline. Total RNA was isolated from harvested trophozoites using trizol reagent (Invitrogen, Carlsbad, CA, U.S.A.) according to the manufacturer's protocol. The RNA was quantified and checked for purity by comparison of absorbance at 260 and 280 nm in the nanodrop spectrophotometer (Thermo Scientific, Wilmington, DE, USA). Integrity of isolated RNA was verified by using Bio-Rad's automated electrophoresis system experion (RNA StdSens analysis kit). All reagents and protocols followed those described in the affymetrix manuals. Briefly, total RNA (5 µg) was reverse transcribed using T7-Oligo (dT) primer in the first strand cDNA synthesis. After second strand synthesis, the double-stranded cDNA template was used for in vitro transcription, in the presence of biotinylated nucleotides to produce labelled cRNA. The cRNA was purified, quantified, fragmented, and hybridized for 16 h at 45°C to custom-generated affymetrix platform microarray (49-7875) with probe sets consisting of 11 probe pairs representing 9,327 *E. histolytica* (Eh_Eia520620F_Eh) and 12,385 *E. invadens* open reading frames (Eh_Eia520620F_Ei). After hybridization, the arrays were washed and stained with streptavidin–phycoerythrin using a GeneChip® Fluidics Station 450 (Affymetrix, Santa Clara, CA, USA), according to the recommendations of the manufacturer. After washing and staining, the GeneChip® arrays were then scanned using the Hewlett-Packard Affymetrix Scanner 3000 (Affymetrix, Santa Clara, CA, USA), and the probe intensities were extracted using Affymetrix® GeneChip® Command Console™ (Affymetrix, Santa Clara, CA, USA).

### Analysis of microarray data

A minimum of two arrays were used for each condition and each time point. Raw Mas5 gene expression data were imported into the GeneSpring GX 10.0.2 program and normalized expression values for each probe set were obtained from raw probe intensities in R 2.7.0 downloaded from the BioConductor project (http://www.bioconductor.org) using robust multiarray averaging with correction for oligosequence (gcRMA). Standard correlation coefficients were calculated using GeneSpring GX 10.0.2 [74]. The data presented in this publication have been deposited in NCBIs Gene Expression Omnibus (GEO, http://www.ncbi.nlm.nih.gov/geo/) and are accessible through GEO Series accession number GSE33312.

### Statistical Analysis

For each experimental condition, two independent biological replicates were made and for each biological replicate, three technical replicates were made. All data are shown as means ± S.D. for the indicated number of experiments. Statistical comparisons were made by Student's t test. Heat maps of metabolite levels were generated using hierarchical clustering based on Pearson correlation coefficients using the MultiExperiment Viewer (MeV) software (Institute for Genomic Research [20]). The Pearson correlation coefficients and their *P*-values (two-tailed) were calculated using GraphPad Prism version 5.04 (GraphPad Software, Inc., San Diego, CA). For microarray data one way ANOVA analysis with Tukey's post hoc test was performed to extract differentially expressed genes. The P-values were calculated using Welch's test, and were corrected by Benjamini-Hochberg method (Dataset S2).

## Supporting Information

Dataset S1 **The quantitative metabolites data during encystation.** Normalized average metabolites data of two biological replicate at 0, 0.5, 2, 8, 24, 48 and 120 h are shown. SD, standard deviation of means; N.D, the metabolite concentrations were below the detection limit of the analysis. All the P values were evaluated by the student's t-test.
(XLSX)

Dataset S2 **Transcriptomic data analyzed in this study.**
(XLSX)

Figure S1 **Heat map representation of microarray expression data of genes involved in glycolysis, serine/cysteine, chitin, glycogen, and polyamine pathways, with the fold change in expression relative to time 0 h at each time point being indicated by different colors boxes.** The color scale is also shown below.
(TIF)
