# Supplementary figures and images for: Metabolic Profiling of the Protozoan Parasite Entamoeba invadens Revealed Activation of Unpredicted Pathway during Encystation
Source: PLoS One. 2012 May 25;7(5):e37740. doi: 10.1371/journal.pone.0037740 (PMC3360610; doi:10.1371/journal.pone.0037740)

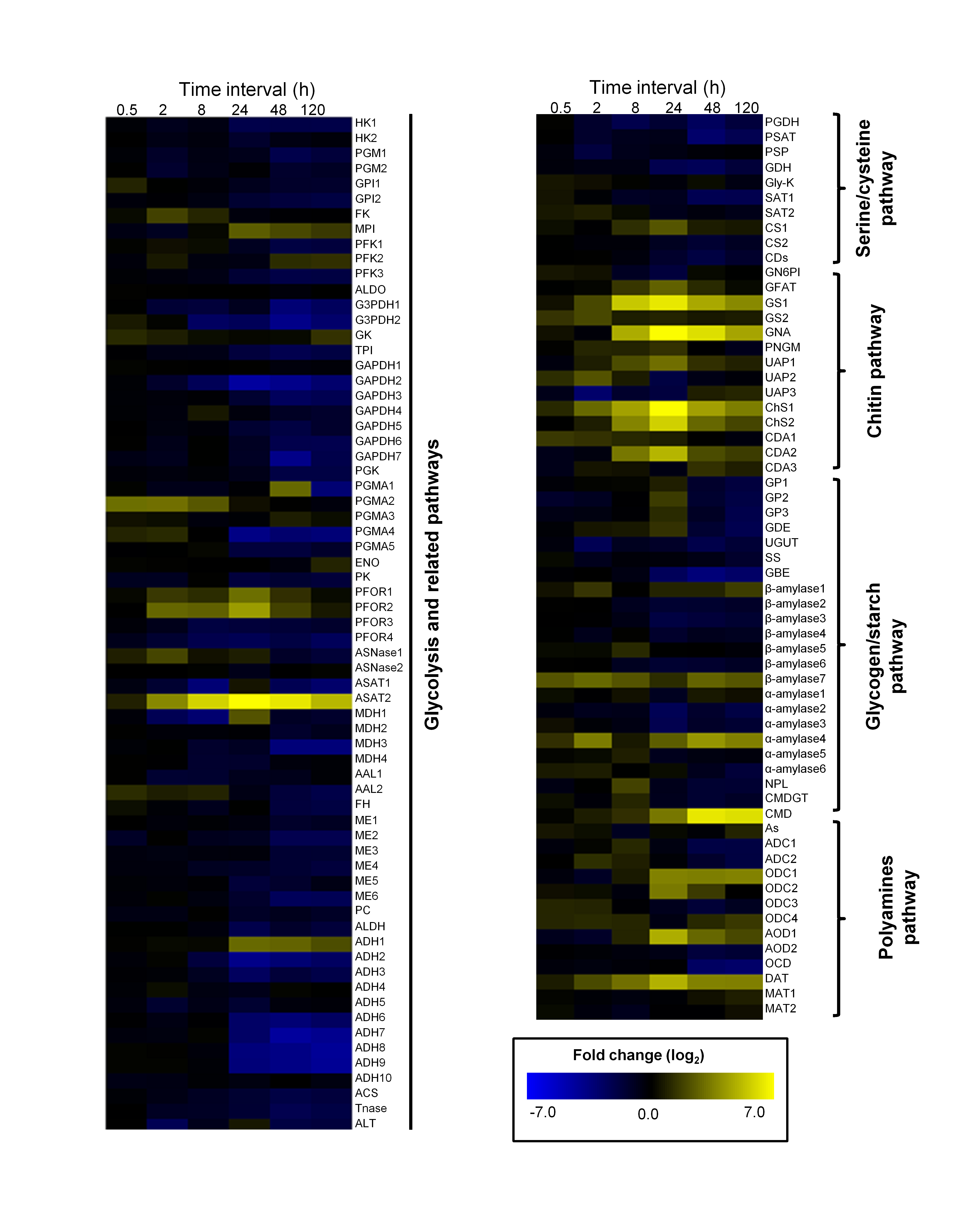

Supplement: Figure S1 — Heat map representation of microarray expression data of genes involved in glycolysis, serine/cysteine, chitin, glycogen, and polyamine pathways, with the fold change in expression relative to time 0 h at each time point being indicated by different colors boxes. The color scale is also shown below. (TIF) [file pone.0037740.s003.tif]
